# Biological activities of phthalocyanines--XVI. Tetrahydroxy- and tetraalkylhydroxy zinc phthalocyanines. Effect of alkyl chain length on in vitro and in vivo photodynamic activities.

**DOI:** 10.1038/bjc.1993.222

**Published:** 1993-06

**Authors:** R. W. Boyle, C. C. Leznoff, J. E. van Lier

**Affiliations:** MRC Group in the Radiation Sciences, Faculty of Medicine, University of Sherbrooke, Quebec, Canada.

## Abstract

Zinc phthalocyanine substituted with four hydroxyl groups attached to the macrocycle, either directly or via spacer chains of three or six carbon atoms, were tested for their photodynamic ability to inactivate Chinese hamster lung fibroblasts (line V-79) in vitro, and to induce regression of EMT-6 tumours grown subcutaneously in Balb/c mice. Their potential to inflict direct cell killing during photodynamic therapy was investigated by examining vascular stasis immediately following photoirradiation using fluorescein as a marker, and also by an in vivo/in vitro EMT-6 cell survival assay. Both of the tetraalkylhydroxy substituted zinc phthalocyanines are effective photodynamic sensitisers in vivo with the tetrapropylhydroxy compound exhibiting about twice the activity of the tetrahexylhydroxy analogue. The differences in activities were accentuated in vitro, the tetrapropylhydroxy compound was two orders of magnitude more potent than the tetrahexylhydroxy analogue in photoinactivating V-79 cells. The tetrahydroxy compound lacking spacer chains failed to exhibit photodynamic activity in either system. Tumour response with the active compounds was preceded by vascular stasis immediate following irradiation which suggests, together with the absence of activity in the in vivo/in vitro assay, that tumour regression involves an indirect response to the photodynamic action rather than direct cell killing. These data demonstrate the importance of the spatial orientation of functional groups around the macrocycle of photosensitisers for their efficacy in the photodynamic therapy of cancer.


 © Macmillan Press Ltd., 1993

### Biological activities of phthalocyanines – XVI. Tetrahydroxy- and tetraalkylhydroxy zinc phthalocyanines. Effect of alkyl chain length on *in vitro* and *in vivo* photodynamic activities

R.W. Boyle[1], C.C. Leznoff[2] & J.E. van Lier[1]

[1]*MRC Group in the Radiation Sciences, Faculty of Medicine, University of Sherbrooke, Quebec, Canada J1H 5N4;* [2]*Department of Chemistry, York University, North York, Ontario, Canada M3J 1P3.*

**Summary** Zinc phthalocyanine substituted with four hydroxyl groups attached to the macrocycle, either directly or via spacer chains of three or six carbon atoms, were tested for their photodynamic ability to inactivate Chinese hamster lung fibroblasts (line V-79) *in vitro*, and to induce regression of EMT-6 tumours grown subcutaneously in Balb/c mice. Their potential to inflict direct cell killing during photodynamic therapy was investigated by examining vascular stasis immediately following photoirradiation using fluorescein as a marker, and also by an *in vivo/in vitro* EMT-6 cell survival assay. Both of the tetraalkylhydroxy substituted zinc phthalocyanines are effective photodynamic sensitisers *in vivo* with the tetrapropylhydroxy compound exhibiting about twice the activity of the tetrahexylhydroxy analogue. The differences in activities were accentuated *in vitro*, the tetrapropylhydroxy compound was two orders of magnitude more potent than the tetrahexylhydroxy analogue in photoinactivating V-79 cells. The tetrahydroxy compound lacking spacer chains failed to exhibit photodynamic activity in either system. Tumour response with the active compounds was preceded by vascular stasis immediate following irradiation which suggests, together with the absence of activity in the *in vivo/in vitro* assay, that tumour regression involves an indirect response to the photodynamic action rather than direct cell killing. These data demonstrate the importance of the spatial orientation of functional groups around the macrocycle of photosensitisers for their efficacy in the photodynamic therapy of cancer.

Photodynamic therapy (PDT) (Dougherty, 1987; Henderson & Dougherty, 1992; Moan & Berg, 1992) of solid tumours is in phase III clinical trials and is soon likely to be an established treatment complementing radio- and chemotherapy. The photosensitiser currently used, Photofrin II™ (P-II), is a complex mixture of porphyrin dimers and oligomers, the active component of which, as yet, remains unidentified. It is, therefore, of increasing importance that 'second generation' photosensitisers of known composition and increased photodynamic activity are developed to provide an alternative to P-II. Phthalocyanines (Pc's) have been proposed as promising potential PDT agents (for recent reviews see van Lier, 1990; Rosenthal, 1991) primarily because of the high molar absorptivity of these compounds in the red region of the visible spectrum ($\varepsilon \approx 10^5\,M^{-1}\,cm^{-1}$ at 670–680 nm when fully monomerised compared to $\varepsilon \simeq 10^3\,M^{-1}\,cm^{-1}$ at 630 nm for P-II) where tissue transmission by visible light is more efficent. Their photodynamic activities *in vivo* largely depend on pharmacokinetics and intratissular distribution pattern and only limited structure-activity relationships have been put forth (Paquette & van Lier, 1992). Degree of sulfonation of Pc's is the most studied structural variable, however, many alternative substituents are possible and remain unexplored. An approach towards the synthesis of novel Pc's on the basis of known structural data, and good photodynamic properties of related compounds, could be potentially rewarding, and this is the rationale for the current study.

Berenbaum *et al.* (1986) tested the *ortho*, *meta* and *para* isomers of *meso*-tetra(hydroxyphenyl)porphine (THPP) for their *in vivo* photodynamic activity and skin photosensitisation. Although the *m*- and *o*-THPP have very similar photophysical properties and similar absorbances at the wavelength used for PDT (Bonnett *et al.*, 1988), the *m*-THPP is twice as effective as *o*-THPP in sensitising tumours, while *o*-THPP is four times as potent as *m*-THPP in sensitising skin. Doses of *p*-THPP and *m*-THPP that produce equal tumour necrosis are of the ratio 5:1, while their molar absorbances at the illuminating wavelengths are of the ratio 2:1. These data suggest that *m*-THPP has considerably greater ability than the *para* and *ortho* isomers to localise at the appropriate site, indicating that the relative special orientation of the hydroxy groups around the macrocycle is of crucial importance. On such accounts we synthesised zinc phthalocyanines (ZnPc) substituted with hydroxyl groups fused directly, and rigidly, to the Pc skeleton (i.e. tetrahydroxy zinc phthalocyanine, $ZnPc(OH)_4$), and with hydroxy groups attached to the Pc by aliphatic chains of three and six carbons respectively (tetrapropylhydroxy zinc phthalocyanine, $ZnPc(prOH)_4$ and tetrahexylhydroxy zinc phthalocyanine, $ZnPc(hxOH)_4$) (Figure 1). Reddi *et al.* (1990) showed that unsubstituted ZnPc is photodynamically active when incorporated in liposomes or low density lipoprotein. The present series of ZnPc derivatives allowed us to study the effect of increasing distances and freedom of movement for the four hydroxyl groups on photodynamic activities both under *in vitro* and *in vivo* conditions. Certain aspects of the mechanism of action were also investigated using an *in vivo/in vitro* assay, and the assessment of vascular occlusion immediately following PDT.

### Materials and methods

### *Photosensitisers*

Synthesis of $ZnPc(OH)_4$ has been described in detail elsewhere (Rosenthal *et al.*, 1987). The $ZnPc(prOH)_4$ and ZnPc $(hxOH)_4$ were prepared from 4-iodophthalonitrile (Marccuccio *et al.*, 1985) by palladium catalysed alkynation, followed by catalytic hydrogenation over 10% palladium on charcoal to give 4-(propylhydroxy)phthalonitrile and 4-(hexylhydroxy)-phthalonitrile, both of which condensed cleanly with zinc metal at 170°C to give $ZnPc(prOH)_4$ and $ZnPc(hxOH)_4$ respectively. The final products were characterised by their absorbance and mass spectra. $ZnPc(prOH)_4$: $\lambda_{max}$ (log ε) = 675 nm (5.48); FAB-MS, $M^+$ (%) = 810 (100). $ZnPc(hxOH)_4$: $\lambda_{max}$ (log ε) = 673 nm (5.4); FABS-MS, $M^+$ (%) = 978 (100). Full details of these syntheses will be published elsewhere. Photofrin II™ was obtained from Quadralogic Technologies Inc., Vancouver, BC, Canada. Tetrasulphonated zinc phthalocyanine ($ZnPcS_4$) was prepared by the condensation method, as previously described (Ali *et al.*, 1988).

Correspondence: J.E. van Lier.
Received 8 July 1992; and in revised form 8 December 1992.

**Figure 1** Chemical structures *m*-THPP **a**, $ZnPc(OH)_4$ (**b**: x = 0), $ZnPc(prOH)_4$ (**b**: x = 3) and $ZnPc(hxOH)_4$ (**b**: x = 6).

### *Animal experiments*

Animal experiments were conducted following the recommendations of the Canadian Council on Animal Care and of an in-house ethics committee. The animals were allowed free access to water and food throughout the experiments. Female Balb/c mice had one tumour transplanted in to the right hind thigh by intradermal injection of $2 \times 10^5$ EMT-6 mouse mammary tumour cells (obtained from Dr C.-W. Lin, Massachusetts General Hospital, Boston) suspended in 0.05 ml of Waymouths' medium (Gibco) (Brasseur *et al.*, 1988). Mice were used 6–7 days post-inoculation when tumours had reached a diameter of 3–5 mm. Mice were injected, via the tail vein, with Pc and P-II in a solution of Cremophore EL (Sigma), propane-1,2-diol, and saline (10:3:87). Sulfonated Pc have been shown to reach optimal concentrations in various rodent tumours at 24–48 h post-injection (Tralau *et al.*, 1987; Rousseau *et al.*, 1990) and we assumed similar biodistribution pattern for the novel Pc derivatives. After 24 h the tumour was irradiated with 650–700 nm light (400 J $cm^{-2}$ at a fluence rate of 180 mW $cm^{-2}$) delivered by a 1000 W Xenon lamp fitted with 10 cm water filter, and LS-700 (Corion) and 2-58 (Corning) filters. In the case of P-II a band of 600–650 nm was used at the same fluence, and fluence rate, using LS-600 (Corion) and 650-FLO7-50 (Ealing) filters. Light was focused on the tumour with lenses to give a final beam of 8 mm in diameter. The fluence at the surface of the tumour was calculated from the area under the absorption peaks for the monomeric dye solutions in methanol. Tumour temperature was measured (Brasseur *et al.*, 1987) and rose to 35°C externally and 32°C internally after 10 min, in both cases the temperature remained constant for the remainder of the irradiation time. Tumour response was assessed qualitatively and followed from initial necrosis (within 24 h), to cure, and for a follow-up period of 30 days. Tumour cure was defined as necrosis of the tumour within 48 h, followed by regrowth of normal tissue in the treatment area and no recurrence of the neoplasm up to 30 days post irradiation. Nine mice were used to confirm the minimal dose of dye needed to reach the cure. No spontaneous regression of the tumour was noted in a control group of nine mice over the time course of the experiment.

### In vitro *photocytotoxicity assay*

Cell survival of Chinese hamster lung fibroblasts (line V-79) was determined by a colony forming assay (Brasseur *et al.*, 1985). Plated cells were incubated with 1 ml of dye in medium containing 1% serum for 1 h in the dark at 37°C in 5% $CO_2$. After removal of the dye solution and washing with PBS, cells were refed with growth medium and exposed for 4 min to red light from two 500 W tungsten/halogen lamps (Sylvania FCL) fitted with a refrigerated filter containing aqueous Rhodamine ($OD_{580} = 1.25$) and a red filter (26-4390, Ealing). The fluence over the absorption peaks of the photoactive monomeric dyes was 2.4 J $cm^{-2}$. The cells were then incubated at 37°C in 5% $CO_2$ for 6–7 days. The dye concentration in $\mu$M required for 90% cell mortality, i.e. the extracellular $LD_{90}$, was used to quantify the activity of each dye preparation. Experiments were run at least three times with three dishes for each concentration point.

### In vivo/in vitro *assay*

The procedure is essentially as described by Henderson (1990). Balb/c mice were implanted with two EMT-6 tumours in the hind thighs. When the tumours reached a diameter of 3–5 mm (6–7 days) mice were injected with 10 $\mu$mol $kg^{-1}$ of Pc or 10 mg $kg^{-1}$ of P-II in saline containing 10% Cremophore EL and 3% propane-1,2-diol. Twenty-four hours post-injection of drug animals were sacrificed and the tumours were excised, minced, and enzymatically digested (30 min in $CaCl_2$ 10 mM, proteinase K (Sigma) 6.5 U, micrococcal (Sigma) 3 U, collagenase (Sigma) 17 U, in 10 ml Hank's buffer saline solution). The digested preparation was then filtered through a 200 mesh sieve and centrifuged at 600 *g* for 5 min. Two hundred cells were placed in 6 cm Petri dishes and incubated for 3 h at 37°C in 5% $CO_2$ in Waymouth's culture medium to allow adhesion to the support. Cells were illuminated as described under *in vitro* photocytotoxicity assay. Fluences were calculated from the area under the absorptoin peaks of the monomeric dye in methanol solution. It is evident that during the cell isolation procedure loosely bound dye may detach from the cells resulting in an underestimation of the potential of the dye to inflict direct cell killing during PDT. However, a positive effect in this assay strongly indicates the involvement of a direct photodynamic action on the tumour cells as part of the overall PDT response.

### *Fluorescein exclusion assay*

Animals were prepared as for PDT, but in this case two tumours were grown, one on each hind thigh. The right tumour was irradiated while the left (control) tumour was shielded from light. Immediately following irradiation mice were injected via the tail vein with 2 mg sodium fluorescein in 0.2 ml phosphate buffered saline. After 2 min mice were sacrificed and placed under a longwave UV lamp to visualise areas penetrated by the dye. Any exclusion of fluorescein from the irradiated area of tumour and surrounding tissue, relative to the control, was noted and photographed.

### Results

The novel compounds $ZnPc(OH)_4$, $ZnPc(prOH)_4$ and ZnPc $(hxOH)_4$ were initially tested for their capacity to photoinactivate V-79 cells under *in vitro* conditions. As can be seen from the survival curves in Figure 2, the $ZnPc(prOH)_4$ is greater than two orders of magnitude more active than the ZnPc substituted with four hexylhydroxy ($ZnPc(hxOH)_4$) or sulphonate ($ZnPcS_4$) groups. The derivative substituted with hydroxyl groups without spacer chains shows little activity under our experimental conditions, even at the maximum concentration used (150µM).

The minimum injected dye doses required for 100% cure of the EMT-6 tumours grown in Balb/c mice are shown in Figure 3. Data for P-II and $ZnPcS_4$ are included for comparison and dye doses are expressed in terms of mg $kg^{-1}$ animal body weight to allow for comparison with P-II for which the molecular weight is unknown. $ZnPc(OH)_4$ clearly shows poor photodynamic activity in this system as, even at the highest injected doses (10 µmol $kg^{-1}$; 6.4 mg $kg^{-1}$) no tumour cure could be obtained. $ZnPc(prOH)_4$ induced 100% tumour cure at doses as low as 0.5 µmol $kg^{-1}$ (0.4 mg $kg^{-1}$) representing an improvement of ten times over $ZnPcS_4$ and 25 times over P-II. PDT with $ZnPc(hxOH)_4$ required twice the injected dose of $ZnPc(prOH)_4$ in order to induce the same photodynamic tumour response, i.e. 1 µmol $kg^{-1}$ (1.0 mg $kg^{-1}$). With both compounds $ZnPc(prOH)_4$ and $ZnPc(hxOH)_4$ administered at the minimum dose levels, fluorescein injected immediately after PDT was excluded from the irradiated area, indicating that tumour necrosis is preceded by vascular stasis. Unirradiated control tumours on the same animals showed strong fluorescence. Failure to observe significant photosensitised EMT-6 cell killing *in vitro* after *in vivo* administration of the novel dyes, suggests that a relatively small amount of dye is retained by the tumour cells (Figure 4). Combined with the vascular shut-down observed immediately after PDT these data strongly suggest that tumour necrosis with these dyes results from an initial vascular effect rather than direct tumour cell killing. In contrast, $ZnPcS_4$ scored in the *in vivo/in vitro* assay an $LD_{90}$ of less than 20 J $cm^{-2}$ light dose, indicative of a good potential to inflict direct cell killing during PDT.

**Figure 2** Photocytotoxicity assay for $ZnPc(OH)_4$ (○), ZnPc $(hxOH)_4$ (□), $ZnPcS_4$ (■) and $ZnPc(prOH)_4$ (△) with V-79 cells. Plated cells were incubated with dye solutions in medium containing 1% serum for 1 h in the dark at 37°C. After removal of the dye solution and washing, cells were exposed for 4 min to red light at a fluence of 2.4 J $cm^{-2}$, incubated for 6–7 days whereafter colonies were counted.

**Figure 3** Minimal injected dye doses (mg $kg^{-1}$) required for 100% cure of EMT-6 tumours on Balb/c mice after identical PDT protocols. In the case of $ZnPc(OH)_4$ no tumour cure was observed at the highest dose (10 µmol $kg^{-1}$; 6.4 mg $kg^{-1}$) tested. Dyes were injected i.v. as Cremophore emulsions, 24 h later the tumours were irradiated with red light (400 J $cm^{-2}$, 180 mW $cm^{-2}$; Pc's 650–700 nm; P-II, 600–650 nm) and tumour response assessed qualitatively from initial necrosis (24 h) to cure.

### Discussion

If advantage is to be taken of the favourable absorption characteristics of Pc type molecules in PDT it is important that structure-activity relationships for this class of compound be defined. To date, research conducted in this area has mainly addressed the effect of the degree of sulphonation of Pc's on their biodistribution and photodynamic activity (for a recent review see Paquette and van Lier, 1992). Commercially available aluminium phthalocyanine sulphonate (AlPcS) has most widely been used for biological studies (Tralau *et al.*, 1987; Matthews & Cui, 1990; Bedwell *et al.*, 1991), but consists of a mixture of tetra, tri, di and monosulphonated AlPc with an average sulphonation level of approx-

**Figure 4** *In vivo/in vitro* assay for $ZnPc(OH)_4$ (○), $ZnPc(prOH)_4$ (▲), $ZnPc(hxOH)_4$ (□) and $ZnPcS_4$ (■). EMT-6 cell survival as a function of the light dose *in vitro*, 24 h after i.v. injection of 10 µmol $kg^{-1}$ of sensitiser *in vivo*.

imately 3.2 and all attendant positional isomers. It has been shown however that the lower sulphonated derivatives, and particularly the amphiphilic $AlPcS_{2a}$ (featuring sulphonate groups on adjacent benzo rings of the Pc macrocycle) localise in tumour cells whereas the tetrasulphonated analogue is mainly detected in the stroma of the tumour (Paquette *et al.*, 1988; Peng *et al.*, 1990; 1991). Thus, in spite of a higher overall tumour retention of the tri- and tetrasulphonated derivates as compared to the mono- and disulphonated analogues (Rousseau *et al.*, 1990; Chan *et al.*, 1990), the latter exhibit better photodynamic properties due to their advantageous intratissular tumour distribution and cell penetration. These different activities and localisation pattern also correlate well with the action mechanisms. In the case of the $AlPcS_{2a}$ light and electron microscopy showed that there was a direct, extensive, photo-damaging action on human tumour cells (LOX xenographs) grown in athymic nude mice whereas treatment of $AlPcS_4$ and light resulted initially in a functional vasogenic response and ultimate damage to the vascular structure of the tumour (Peng *et al.*, 1990). Simple procedures to prepare AlPcS enriched in the active disulphonated derivatives have been reported (Ali *et al.*, 1988; Ambroz *et al.*, 1991) and their *in vivo* pattern of photosensitisation have been studied in detail (Nuutinen *et al.*, 1991; Loh *et al.*, 1992). Addition of hydrophobic tertiary butyl (Paquette *et al.*, 1991), phthalimidomethyl (Boyle *et al.*, 1992) or benzene groups (Margaron *et al.*, 1992) on the non-substituted, adjacent benzo rings of disulphonated Pc's further enhances the amphiphilic properties of these drugs resulting in increased potential for direct cell killing during PDT. Differently sulphonated tetraphenylporphins follow similar biodistribution pattern as the analogous sulphophthalocyanines, with the derivative featuring two adjacent sulfonates showing the highest photocytotoxicity (Kessel *et al.*, 1987).

The work of Bonnett *et al.* (1987) with the *meso*-tetra-(hydroxyphenyl)porphins (THPP) yielded structure-activity relationships of particular interest for the development of Pc based photosensitizers. Whereas, the *o*-, *m*- and *p*-isomers of THPP exhibited similar photophysical properties (Bonnett *et al.*, 1988) large differences were noted in their biological activities, with the *m*-isomer showing the highest potency in sensitising tumours *in vivo* (Berenbaum *et al.*, 1986). Unlike the disulphonated phthalocyanines and tetraphenylporphins, the THPP isomers are symmetric, lipophilic molecules lacking the characteristic amphiphilic properties of the disulphonated photosensitisers. They distribute in tumours similarly to the main components of P-II with membranes as the major target and some localisation in the cytoplasm (Peng *et al.*, 1991). These data suggest that an array of four hydroxyl groups arranged judiciously around a rigid photosensitising structure could impart interesting biological activities to our Pc. As it was difficult to ascertain the exact orientation of the hydroxy groups in *m*-THPP, due to the rotational flexibility of the single bond linking the hydroxyphenyl groups to the porphine macrocycle, we synthesised three Pc molecules with distinct differences in the distances between, and flexibility of the hydroxyl groups (Figure 1). Our tumour response results indicated that the spatial arrangement and conformational flexibility of the hydroxyl groups is essential for PDT efficacy, as $ZnPc(OH)_4$ was found to be completely photodynamically inactive, even at $10\,\mu mol\,kg^{-1}$ injected dose (twice the dose at which 100% tumour eradication can be achieved using $ZnPcS_4$). In contrast, tumour cure with $ZnPc(hxOH)_4$ and $ZnPc(prOH)_4$ was reached at doses 5 and 10 times lower than for $ZnPcS_4$ and up to 25 times lower than for P-II. These differences in biological activities are further accentuated under *in vitro* conditions using a population of rapidly dividing V-79 cells, a pattern previously noted with isomeric disulfonated Pc's (Brasseur *et al.*, 1988). The relative photoactivities between the propylhydroxy and hexylhydroxy ZnPc derivatives changes from a factor 2 *in vivo* to a factor $10^2$ under *in vitro* conditions. The $ZnPc(OH)_4$ shows little activity in both our *in vitro* and *in vivo* model. In a pattern similar to the differences in activity between *o*-, *m*- and *p*-THPP we see that in both cases the compounds with hydroxy groups locked rigidly, relative to one another and the macrocycle ($ZnPc(OH)_4$ and *p*-THPP) are the least active, and that allowing some degree of movement to the hydroxy groups increases this activity. Such correlations are relevant to recent theoretical considerations put forth by Winkelman *et al.* (1993) concerning stereochemical requirements for photosensitisers to facilitate transport and binding in biological systems. These authors compared geometrical features of a number of substituted porphyrins and phthalocyanines with published biological activities and suggested that there exists a critical distance of about 1.2 nm between oxygens (sulphonate, carboxyl or hydroxyl substituents) that characterises the biologically active structures for PDT. The flexibility of both our alkylhydroxy ZnPc's will allow confirmations to match such a geometry and particularly the tetrapropylhydroxy derivative has a high number of low energy conformers in which two adjacent OH groups are separated by 1.1–1.2 nm.

Vascular occlusion of the EMT-6 tumours immediately after PDT with both the $ZnPc(prOH)_4$ and $ZnPc(hxOH)_4$ indicates that tumour regression mainly results from indirect photodynamic effects. The absence of photodynamic cell killing in the *in vivo*/*in vitro* assay is in line with such a mechanism and suggest that only low concentrations of these dyes are retained by the tumour cells. This does however not exclude the possible presence of labile-bound dye *in vivo*, providing direct photodynamic cytotoxicity, as seems to be the case with *m*-THPP mediated PDT response (Peng *et al.*, 1991). Although early PDT-induced hypoxia can severely limit the potential for direct cell killing of a dye (Henderson & Fingar, 1989), the ultimate tumour eradication likely depends upon the effective destruction of the microvasculature in the tumour and the surrounding normal tissue (Fingar & Henderson, 1987). Intratissular localisation pattern of *m*-THPP in human melanoma xenografts in nude mice resembles that of P-II (Peng *et al.*, 1991), whereas the alkylhydroxy ZnPc's gave similar response pattern at P-II in our current assays. This suggests that P-II, *m*-THPP, and our novel $ZnPc(prOH)_4$ and $ZnPc(hxOH)_4$ share the capacity to induce similar photodynamic action mechanisms, with the extent of their potential for direct photocytotoxicity *in vivo* depending on the actual PDT conditions used.

In conclusion, we have shown that it is possible to extrapolate structure-activity relationships determined for porphyrin based photosensitisers to other photoactive species with better absorption characteristics. This could allow the considerable data available on structure-activity relationships for porphyrins to be applied ot phthalocyanine based photosensitisers and permit a focus of synthetic efforts for the design of a viable Pc based PDT agent.

This work was supported by the Natural Sciences and Engineering Research Council and the Medical Research Council of Canada.

Abbreviations: Pc, phthalocyanines; PDT, photodynamic therapy; ZnPc, zinc phthalocyanine; $ZnPcS_4$, tetrasulphonated zinc phthalocyanine; $ZnPc(OH)_4$, tetrahydroxy zinc phthalocyanine; $ZnPc(prOH)_4$, tetrapropylhydroxy zinc phthalocyanine; $ZnPc(hxOH)_4$, tetrahexylhydroxy zinc phthalocyanine; P-II, Photofrin II™.

### References

ALI, H., LANGOIS, R., WAGNER, J.R., BRASSEUR, N., PAQUETTE, B. & VAN LIER, J.E. (1988). Biological activities of phthalocyanines-X. Synthesis and analyses of sulfonated phthalocyanines. *Photochem. Photobiol.*, **47**, 713–717.

AMBROZ, M., BEEBY, A., MACROBERT, A.J., SIMPSON, M.S.C., SVENSEN, R.K. & PHILLIPS, D. (1991). Preparation, analytical and fluorescence spectroscopic studies of sulphonated aluminium phthalocyanine photosensitisers. *J. Photochem. Photobiol. B. Biol.*, **9**, 87–95.

BEDWELL, J., CHATLANI, P.T., MACROBERT, A.J., ROBERTS, J.E., BARR, H., DILLON, J. & BOWN, S.G. (1991). Enhanced tumour selectivity of photodynamic therapy in the rat colon using a radioprotection agent. *Photochem. Photobiol.*, **53**, 753–756.

BERENBAUM, M.C., AKANDE, S.L., BONNETT, R., KAUR, H., IOANNOU, S., WHITE, R.D. & WINFIELD, U.-J. (1986). *meso*-Tetra-(hydroxyphenyl)porphyrins, a new class of potent tumour photosensitisers with favourable selectivity. *Br. J. Cancer*, **54**, 717–725.

BONNETT, R., IOANNOU, S., WHITE, R.D., WINFIELD, U.-J. & BERENBAUM, M.C. (1987). *meso*-Tetra(hydroxyphenyl)porphyrins as tumour photosensitizers: chemical and photochemical aspects. In *From Photophysics to Photobiology*. Favre, A. Tyrrell, R. & Cadet, J. (eds), *Photobiochem. Photobiophys.*, Suppl. pp. 45–46. Elsevier: Amsterdam.

BONNETT, R., MCGARVEY, D.J., HARRIMAN, A., LAND, E.J., TRUSCOTT, T.G. & WINFIELD, U.-J. (1988). Photophysical properties of *meso*-tetraphenylporphin and some *meso*-tetra(hydroxyphenyl)-porphyrins. *Photochem. Photobiol.*, **48**, 271–276.

BOYLE, R.W., PAQUETTE, B. & VAN LIER, J.E. (1992). Biological activities of phthalocyanines. XIV. Effect of hydrophobic phthalimidomethyl groups on the *in vivo* phototoxicity and mechanism of photodynamic action of sulphonated aluminium phthalocyanines. *Br. J. Cancer*, **65**, 813–817.

BRASSEUR, N., ALI, H., AUTENRIETH, D., LANGLOIS, R. & VAN LIER, J.E. (1985). biological activities of phthalocyanines. III. Photoinactivation of V-79 Chinese hamster cells by tetrasulfophthalocyanines. *Photochem. Photobiol.*, **42**, 515–521.

BRASSEUR, N., ALI, H., LANGLOIS, R. & VAN LIER, J.E. (1988). Biological activities of phthalocyanines. IX. Photosensitization of V-79 Chinese hamster cells and EMT-6 mouse mammary tumor by selectivity sulfonated zinc phthalocyanines. *Photochem. Photobiol.*, **47**, 705–711.

BRASSEUR, N., ALI, H., LANGLOIS, R., WAGNER, R.J., ROUSSEAU, J. & VAN LIER, J.E. (1987). Biological activities of phthalocyanines. V. Photodynamic therapy of EMT-6 mammary tumors in mice with sulfonated phthalocyanines. *Photochem. Photobiol.*, **45**, 581–586.

CHAN, W.-S., MARSHALL, J.F., SVENSEN, R., BEDWELL, J. & HART, I.R. (1990). Effect of sulfonation on the cell and tissue distribution of the photosensitizer aluminium phthalocyanine. *Cancer Res.*, **50**, 4533–4538.

CHATLANI, P.T., BEDWELL, J., MACROBERTS, A.J., BARR, H., BOULOS, P., KRASNER, N., PHILLIPS, D. & BOWN, S.G. (1991). Comparison of distribution and photodynamic effects of di- and tetra-sulfonated aluminum phthalocyanines in normal rat colon. *Photochem. Photobiol.*, **53**, 745–751.

DOUGHERTY, T.J. (1987). Photosensitizers: therapy and detection of malignant tumors. *Photochem. Photobiol.*, **45**, 897–889.

FINGAR, V.H. & HENDERSON, B.W. (1987). Drug and light dose dependence of photodynamic therapy: a study of tumor and normal tissue response. *Photochem. Photobiol.*, **46**, 837–841.

HENDERSON, B.W. (1990). Probing the effects of photodynamic therapy through *in vivo-in vitro* methods. In *Photodynamic Therapy of Neoplastic Disease, Vol. 1.* Kessel, D. (ed.), pp. 169–188, CRC Press: Boca Raton, FL.

HENDERSON, B.W. & DOUGHERTY, T.J. (1992). How does photodynamic therapy work? *Photochem. Photobiol.*, **55**, 145–157.

HENDERSON, B.W. & FINGAR, V.H. (1989). Oxygen limitation of direct tumor cell kill during photodynamic treatment of a murine tumor model. *Photochem. Photobiol.*, **49**, 299–304.

KESSEL, D., THOMPSON, P., SAATIO, K. & NANTWI, K.D. (1987). Tumor localisation and photosensitization by sulfonated derivates of tetraphenylporphin. *Photochem. Photobiol.*, **45**, 787–790.

LOH, C.S., BEDWELL, J., MACROBERT, A.J., KRASNER, N., PHILLIPS, D. & BOWN, S.G. (1992). Photodynamic therapy of the normal rat stomach: a comparative study between di-sulphonated aluminium phthalocyanine and 5-aminolaevulinic acid. *Br. J. Cancer*, **66**, 452–462.

MARCCUCCIO, S.M., SVIRSKAYA, P.I., GREENBERG, S., LEVER, A.B.P. & LEZNOFF, C.C. (1985). Binuclear phthalocyanines covalently linked through two and four atom bridges. *Can. J. Chem.*, **63**, 623–631.

MARGARON, P., LANGLOIS, R., VAN LIER, J.E. & GASPARD, S. (1992). Photodynamic properties of naphthosulfobenzoporphyrazines, novel asymmetric, amphiphilic phthalocyanine derivatives. *J. Photochem. Photobiol. B. Biol.*, **14**, 187–199.

MATTHEWS, E.K. & CUI, Z.J. (1990). Photodynamic action of sulphonated aluminium phthalocyanines (SALPC) on AR4-2J cells, a carcinoma cell line of rat exocrine pancreas. *Br. J. Cancer*, **61**, 695–701.

MOAN, J. & BERG, K. (1992). Photochemotherapy of cancer: experimental research. *Photochem. Photobiol.*, **55**, 931–948.

NUUTINEN, P.J.O., CHATLANI, P.T., BEDWELL, J., MACROBERT, A.J., PHILLIPS, D. & BOWN, S.G. (1991). Distribution and photodynamic effect of di-sulphonated aluminium phthalocyanine in the pancreas and adjacent tissues in the Syrian golden hamster. *Br. J. Cancer*, **64**, 1108–1115.

PAQUETTE, B., ALI, H., LANGLOIS, R. & VAN LIER, J.E. (1988). Biological activities of phthalocyanines. VIII. Cellular distribution in V-79 Chinese hamster cells and phototoxicity of selectively sulfonated aluminium phthalocyanines. *Photochem. Photobiol.*, **47**, 215–220.

PAQUETTE, B., ALI, H. & VAN LIER, J.E. (1991). Phtalocyanines pour la thérapie photodynamique du cancer: effet des substituants tertio-butyles sur l'accumulation cellulaire et l'activité photodynamique de phtalocyanines sulfonées de gallium. *J. Chim. Phys.*, **88**, 1113–1123.

PAQUETTE, B. & VAN LIER, J.E. (1992). Phthalocyanines and related compounds: structure-activity relationships. In *Photodynamic Therapy: Basic Principles and Clinical Aspects*. Henderson, B.W. & Dougherty, T.J. (eds), pp. 145–156. Marcel Dekker: New York, NY.

PENG, Q., MOAN, J., FARRANTS, G., DANIELSEN, M.E. & RIMINGTON, C. (1991). Localization of potent photosensitizers in human tumor LOX, by means of laser scanning microscopy. *Cancer Lett.*, **58**, 17–27.

PENG, Q., MOAN, J., NESLAND, J.M. & RIMINGTON, C. (1990). Aluminium phthalocyanines with asymmetrical lower sulfonation and with symmetrical higher sulfonation: a comparison of localizing and photosensitizing mechanism in human tumor LOX xenografts. *Int. J. Cancer*, **46**, 719–726.

REDDI, E., ZHOU, C., BIOLO, R., MENEGALDO, E. & JORI, G. (1990). Liposome- or LDL-administered Zn(II)-phthalocyanine as a photodynamic agent for tumours. I. Pharmacokinetic properties and phototherapeutic efficiency. *Br. J. Cancer*, **61**, 407–411.

ROSENTHAL, I. (1991). Phthalocyanines as photodynamic sensitizers. *Photochem. Photobiol.*, **53**, 859–870.

ROSENTHAL, I., BEN-HUR, E., GREENBERG, S., CONCEPCION-LAM, A., DREW, D.M. & LEZNOFF, C.C. (1987). The effect of substituents on phthalocyanine phototoxicity. *Photochem. Photobiol.*, **46**, 959–963.

ROUSSEAU, J., LANGLOIS, R., ALI, H. & VAN LIER, J.E. (1990). Biological activities of phthalocyanines. XII. Synthesis, tumor uptake and biodistribution of $^{14}C$-labeled di- and trisulfonated gallium phthalocyanine in $C_3H$ mice. *J. Photochem. Photobiol. B. Biol.*, **6**, 121–132.

TRALAU, C.J., BARR, H., SANDEMAN, D.R., BARTON, T., LEWIN, M.R. & BOWN, S.G. (1987). Aluminium sulfonated phthalocyanine distribution in rodent tumours of the colon, brain and pancreas. *Photochem. Photobiol.*, **46**, 777–781.

VAN LIER, J.E. (1990). Phthalocyanines as sensitizers for PDT of cancer. In *Photodynamic Therapy of Neoplastic Disease*, Vol. 1. Kessel, D. (ed.) pp. 279–290. CRC Press: Boca Raton, FL.

WINKELMAN, J.W., ARAD, D. & KIMEL, S. (1993). Stereochemical factors in the transport and binding of photosensitisers in biological systems and in photodynamic therapy. *J. Photochem. Photobiol. B. Biol.* (in press).


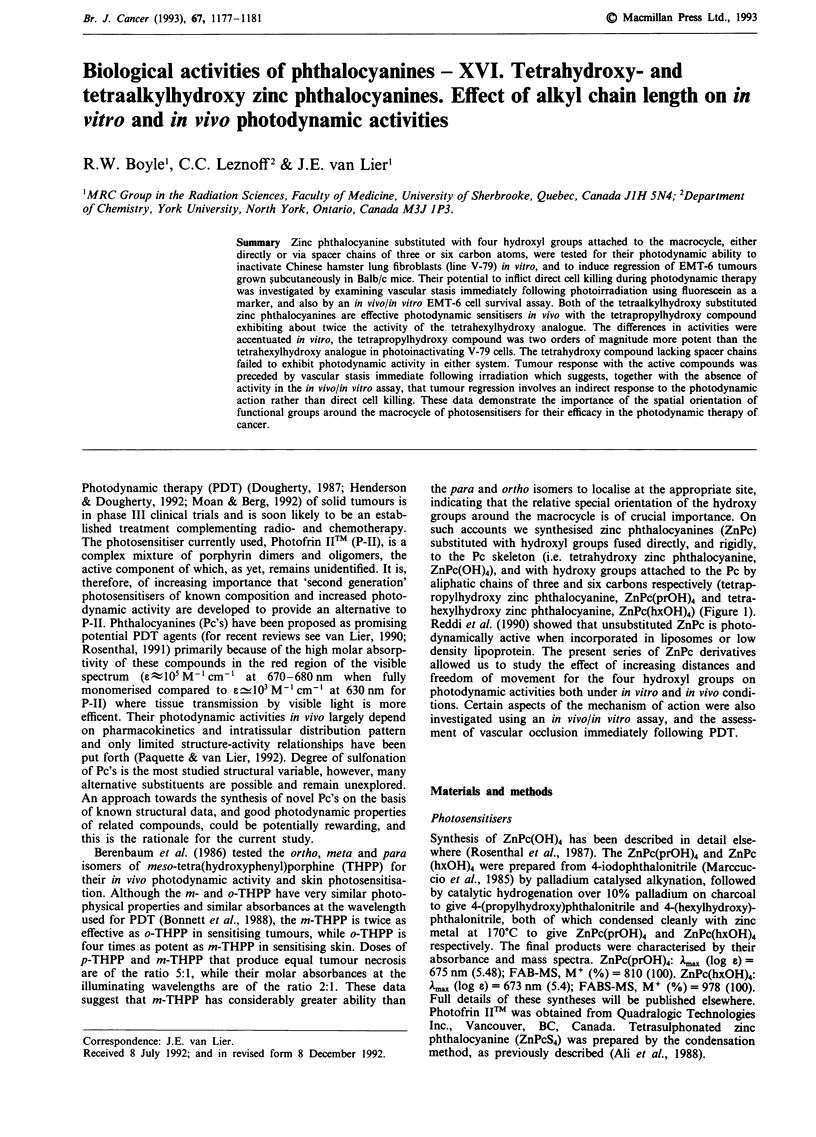

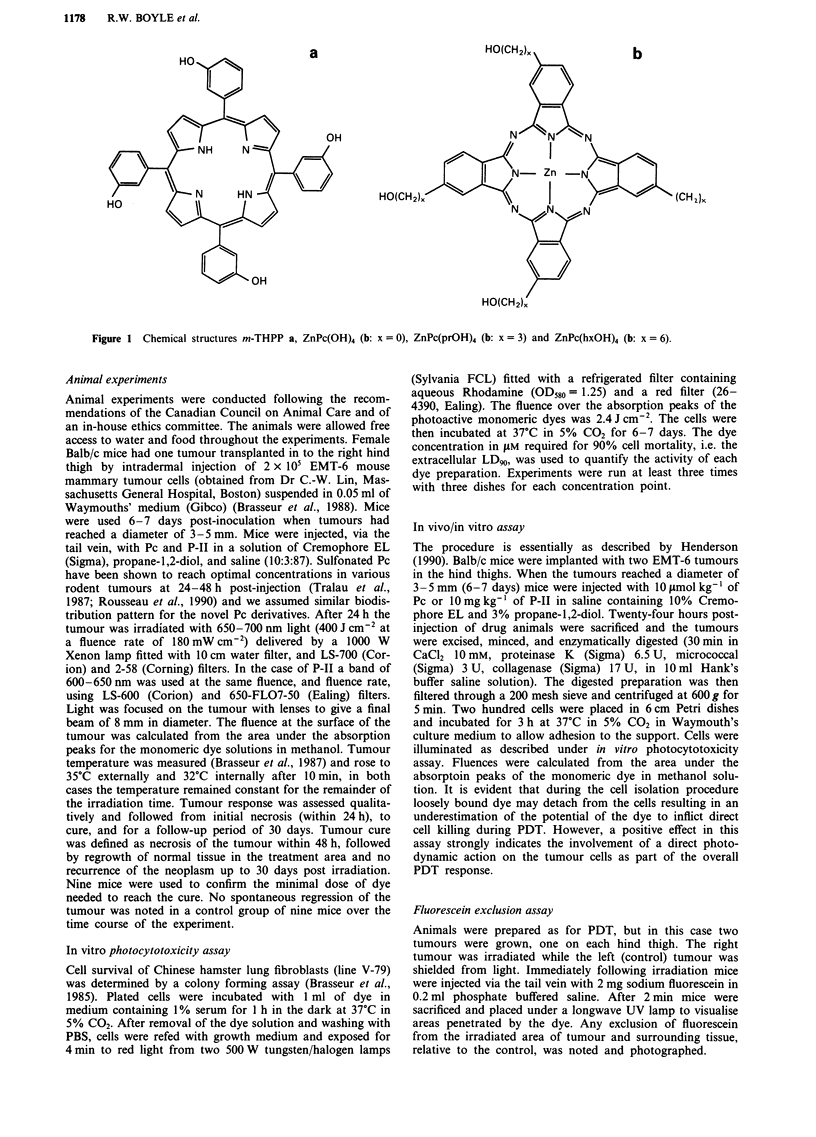

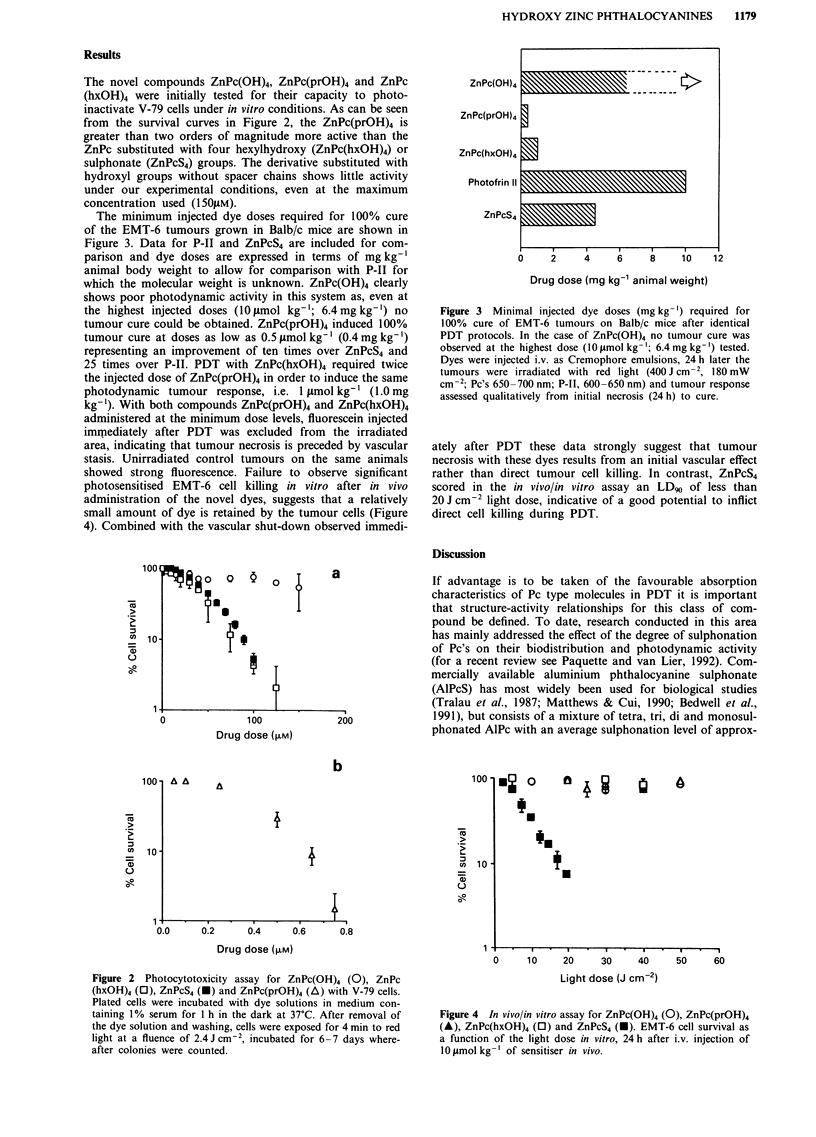

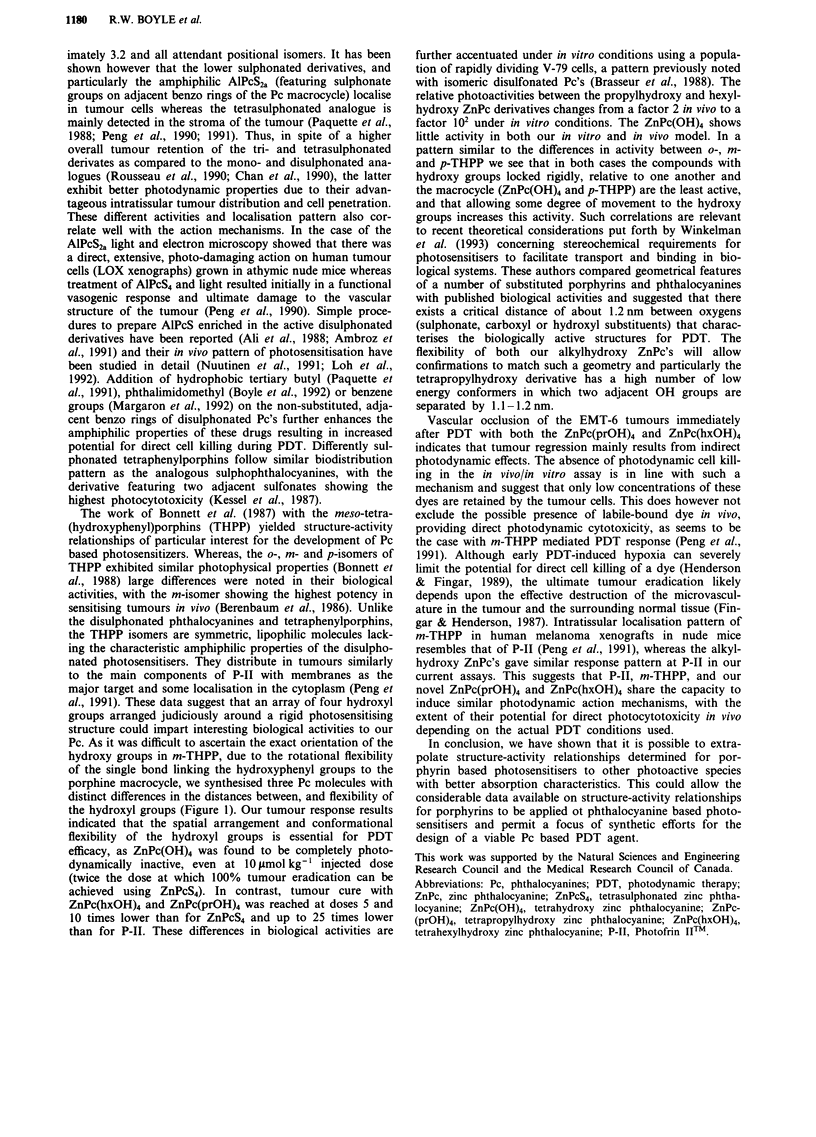

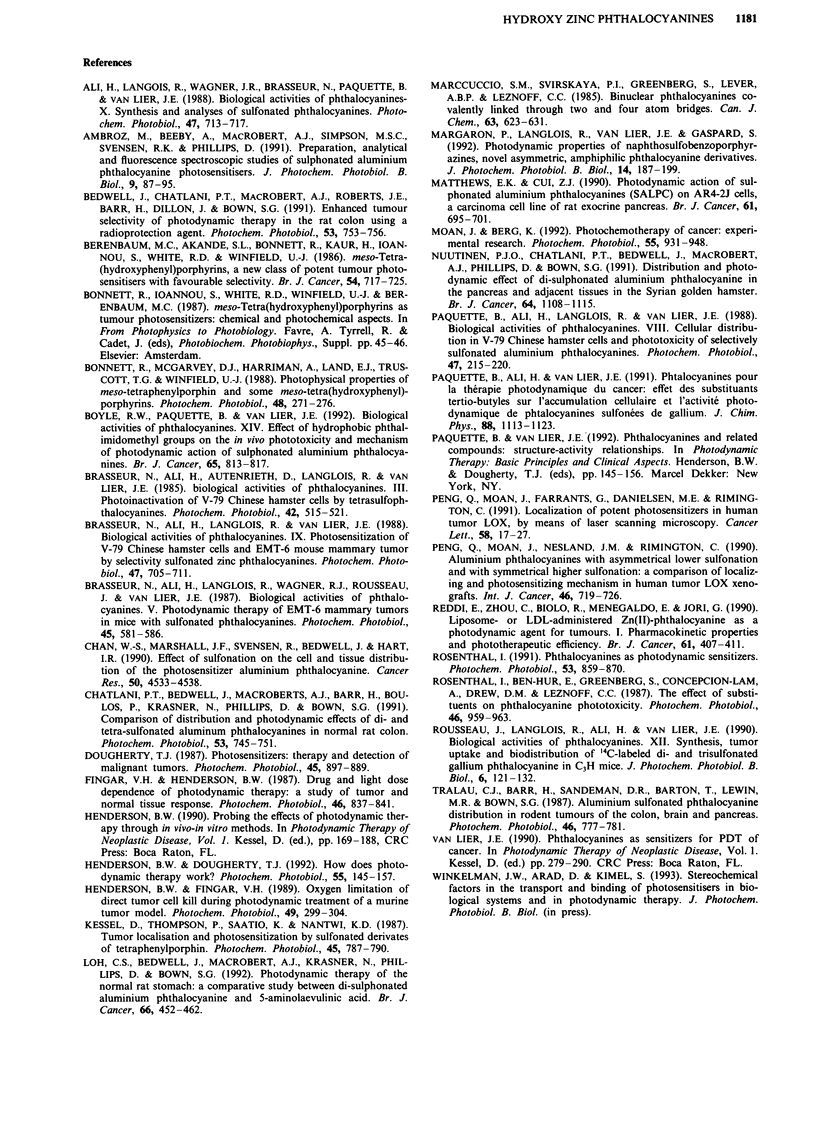


## References

[OCR_00562] Ali H., Langlois R., Wagner J. R., Brasseur N., Paquette B., van Lier J. E. (1988). Biological activities of phthalocyanines--X. Syntheses and analyses of sulfonated phthalocyanines.. Photochem Photobiol.

[OCR_00568] Ambroz M., Beeby A., MacRobert A. J., Simpson M. S., Svensen R. K., Phillips D. (1991). Preparative, analytical and fluorescence spectroscopic studies of sulphonated aluminium phthalocyanine photosensitizers.. J Photochem Photobiol B.

[OCR_00575] Bedwell J., Chatlani P. T., MacRobert A. J., Roberts J. E., Barr H., Dillon J., Bown S. G. (1991). Enhanced tumour selectivity of photodynamic therapy in the rat colon using a radioprotective agent.. Photochem Photobiol.

[OCR_00583] Berenbaum M. C., Akande S. L., Bonnett R., Kaur H., Ioannou S., White R. D., Winfield U. J. (1986). meso-Tetra(hydroxyphenyl)porphyrins, a new class of potent tumour photosensitisers with favourable selectivity.. Br J Cancer.

[OCR_00596] Bonnett R., McGarvey D. J., Harriman A., Land E. J., Truscott T. G., Winfield U. J. (1988). Photophysical properties of meso-tetraphenylporphyrin and some meso-tetra(hydroxyphenyl)porphyrins.. Photochem Photobiol.

[OCR_00600] Boyle R. W., Paquette B., van Lier J. E. (1992). Biological activities of phthalocyanines. XIV. Effect of hydrophobic phthalimidomethyl groups on the in vivo phototoxicity and mechanism of photodynamic action of sulphonated aluminium phthalocyanines.. Br J Cancer.

[OCR_00607] Brasseur N., Ali H., Autenrieth D., Langlois R., van Lier J. E. (1985). Biological activities of phthalocyanines--III. Photoinactivation of V-79 Chinese hamster cells by tetrasulfophthalocyanines.. Photochem Photobiol.

[OCR_00620] Brasseur N., Ali H., Langlois R., Wagner J. R., Rousseau J., van Lier J. E. (1987). Biological activities of phthalocyanines--V. Photodynamic therapy of EMT-6 mammary tumors in mice with sulfonated phthalocyanines.. Photochem Photobiol.

[OCR_00613] Brasseur N., Ali H., Langlois R., van Lier J. E. (1988). Biological activities of phthalocyanines--IX. Photosensitization of V-79 Chinese hamster cells and EMT-6 mouse mammary tumor by selectively sulfonated zinc phthalocyanines.. Photochem Photobiol.

[OCR_00627] Chan W. S., Marshall J. F., Svensen R., Bedwell J., Hart I. R. (1990). Effect of sulfonation on the cell and tissue distribution of the photosensitizer aluminum phthalocyanine.. Cancer Res.

[OCR_00635] Chatlani P. T., Bedwell J., MacRobert A. J., Barr H., Boulos P. B., Krasner N., Phillips D., Bown S. G. (1991). Comparison of distribution and photodynamic effects of di- and tetra-sulphonated aluminium phthalocyanines in normal rat colon.. Photochem Photobiol.

[OCR_00640] Dougherty T. J. (1987). Photosensitizers: therapy and detection of malignant tumors.. Photochem Photobiol.

[OCR_00644] Fingar V. H., Henderson B. W. (1987). Drug and light dose dependence of photodynamic therapy: a study of tumor and normal tissue response.. Photochem Photobiol.

[OCR_00655] Henderson B. W., Dougherty T. J. (1992). How does photodynamic therapy work?. Photochem Photobiol.

[OCR_00659] Henderson B. W., Fingar V. H. (1989). Oxygen limitation of direct tumor cell kill during photodynamic treatment of a murine tumor model.. Photochem Photobiol.

[OCR_00664] Kessel D., Thompson P., Saatio K., Nantwi K. D. (1987). Tumor localization and photosensitization by sulfonated derivatives of tetraphenylporphine.. Photochem Photobiol.

[OCR_00671] Loh C. S., Bedwell J., MacRobert A. J., Krasner N., Phillips D., Bown S. G. (1992). Photodynamic therapy of the normal rat stomach: a comparative study between di-sulphonated aluminium phthalocyanine and 5-aminolaevulinic acid.. Br J Cancer.

[OCR_00682] Margaron P., Langlois R., van Lier J. E., Gaspard S. (1992). Photodynamic properties of naphthosulfobenzoporphyrazines, novel asymmetric, amphiphilic phthalocyanine derivatives.. J Photochem Photobiol B.

[OCR_00688] Matthews E. K., Cui Z. J. (1990). Photodynamic action of sulphonated aluminium phthalocyanine (SALPC) on AR4-2J cells, a carcinoma cell line of rat exocrine pancreas.. Br J Cancer.

[OCR_00694] Moan J., Berg K. (1992). Photochemotherapy of cancer: experimental research.. Photochem Photobiol.

[OCR_00698] Nuutinen P. J., Chatlani P. T., Bedwell J., MacRobert A. J., Phillips D., Bown S. G. (1991). Distribution and photodynamic effect of disulphonated aluminium phthalocyanine in the pancreas and adjacent tissues in the Syrian golden hamster.. Br J Cancer.

[OCR_00705] Paquette B., Ali H., Langlois R., van Lier J. E. (1988). Biological activities of phthalocyanines--VIII. Cellular distribution in V-79 Chinese hamster cells and phototoxicity of selectively sulfonated aluminum phthalocyanines.. Photochem Photobiol.

[OCR_00728] Peng Q., Moan J., Farrants G., Danielsen H. E., Rimington C. (1991). Localization of potent photosensitizers in human tumor LOX by means of laser scanning microscopy.. Cancer Lett.

[OCR_00732] Peng Q., Moan J., Nesland J. M., Rimington C. (1990). Aluminum phthalocyanines with asymmetrical lower sulfonation and with symmetrical higher sulfonation: a comparison of localizing and photosensitizing mechanism in human tumor LOX xenografts.. Int J Cancer.

[OCR_00739] Reddi E., Zhou C., Biolo R., Menegaldo E., Jori G. (1990). Liposome- or LDL-administered Zn (II)-phthalocyanine as a photodynamic agent for tumours. I. Pharmacokinetic properties and phototherapeutic efficiency.. Br J Cancer.

[OCR_00749] Rosenthal I., Ben-Hur E., Greenberg S., Concepcion-Lam A., Drew D. M., Leznoff C. C. (1987). The effect of substituents on phthalocyanine photocytotoxicity.. Photochem Photobiol.

[OCR_00745] Rosenthal I. (1991). Phthalocyanines as photodynamic sensitizers.. Photochem Photobiol.

[OCR_00755] Rousseau J., Langlois R., Ali H., van Lier J. E. (1990). Biological activities of phthalocyanines. XII: Synthesis tumor uptake and biodistribution of 14C-labeled disulfonated and trisulfonated gallium phthalocyanine in C3H mice.. J Photochem Photobiol B.

[OCR_00762] Tralau C. J., Barr H., Sandeman D. R., Barton T., Lewin M. R., Bown S. G. (1987). Aluminum sulfonated phthalocyanine distribution in rodent tumors of the colon, brain and pancreas.. Photochem Photobiol.

